# Transdermal microneedle patches as a promising drug delivery system for anti-obesogenic molecules

**DOI:** 10.3389/fbioe.2024.1380537

**Published:** 2024-06-11

**Authors:** Calef Sánchez-Trasviña, David Coronel-Meneses, Aleyda Margarita Escobar-Fernández, Karla Mayolo-Deloisa

**Affiliations:** ^1^ Tecnologico de Monterrey, Institute for Obesity Research, Monterrey, NL, Mexico; ^2^ Tecnologico de Monterrey, Escuela de Ingeniería y Ciencias, Centro de Biotecnología-FEMSA, Monterrey, NL, Mexico

**Keywords:** transdermal patches, microneedles, anti-obesogenic treatment, transdermal drug delivery systems, obesity treatment

## Abstract

Obesity, characterized by excessive storage of lipids, has become a global pandemic with high incidence levels, and its forecast is not encouraging. Currently, there are different strategies to treat obesity; however, these conventional methods have various limitations. Lifestyle changes may result in poor outcomes due to the complexity of obesity causes, pharmaceutic treatments produce severe side effects, and bariatric surgery is highly invasive. In the search for alternative treatments to fight obesity, transdermal drug delivery systems of anti-obesogenic molecules have gained particular attention. However, the diffusion of molecules through the skin is the main drawback due to the characteristics of different layers of the skin, principally the *stratum corneum* and its barrier-like behavior. In this sense, microneedles patches (MP) have emerged to overcome this limitation by piercing the skin and allowing drug delivery inside the body. Although MP have been studied for some years, it was not until about 2017 that their potential as anti-obesogenic treatment was reported. This article aims to summarize and analyze the strategies employed to produce MP and to embed the active molecules against obesity. Special attention is focused on the microneedle’s material, geometry, array, and additional delivery strategies, like nanoencapsulation. MP are a promising tool to develop an easy-access treatment, avoiding the digestive tract and with the capacity to enhance the anti-obesogenic activity by delivering one or more active molecules.

## 1 Introduction

Obesity is an imbalance between the intake and expenditure of energy, which produces excessive storage of lipids and, therefore, entails an increment in body mass index (Chang and Hung, 2022). Obesity is related to several metabolic disorders and diseases like type 2 diabetes, cancer, hypertension, ischemic heart disease, sleep apnea, chronic inflammation, insulin resistance, and hyperlipidemia (Dangol et al., 2017; Bao et al., 2021; Zu et al., 2021; Abbasi et al., 2022). Therefore, obesity is considered a global issue that impacts society in different dimensions. In 2019, 5 million deaths globally were attributed to obesity-related diseases. By 2020, 2.6 billion people worldwide were diagnosed with obesity or overweight, and this is projected to increase to 3 billion in 2025. Moreover, the global economic expenses due to overweight and obesity in 2022 were calculated at around USD 2 trillion, and it is estimated to increase to more than USD 4 trillion by 2035 if preventive and corrective measures are not urgently addressed (World Obesity Federation, 2022; Lobstein et al., 2023).

Nowadays, it is common to fight obesity through physical exercise, controlled diets, and lifestyle changes. However, due to the complexity of obesity causes (genetic, environmental, and metabolic), it may result in poor outcomes (Abbasi et al., 2022). Another way to treat obesity is bariatric surgery, which is considered the standard of care and primary part of obesity management by the American Society for Metabolic and Bariatric Surgery (Ariamoghaddam et al., 2018; Thenappan and Nadler, 2019). On the other hand, pharmacotherapy is needed for patients with body mass index (BMI) ≥30 kg/m^2^ or BMI ≥27 kg/m^2^ with obesity-related diseases (Wen et al., 2022). Currently, the FDA-approved drugs to treat obesity are orlistat, phentermine-topiramate ER, naltrexone-bupropion, liraglutide, semaglutide, tirzetapide, and setmelanotide (Yanovski and Yanovski, 2021; Chakhtoura et al., 2023). Despite several drugs having been developed to treat obesity, they have limited efficacy, yet significant side effects such as cardiovascular and cerebrovascular problems, cancer, or psychological problems like depression or suicidal ideation (Müller et al., 2022). In this sense, many efforts have been focused on two aspects: new emerging anti-obesity compounds and strategies for their delivery system.

**FIGURE 1:**
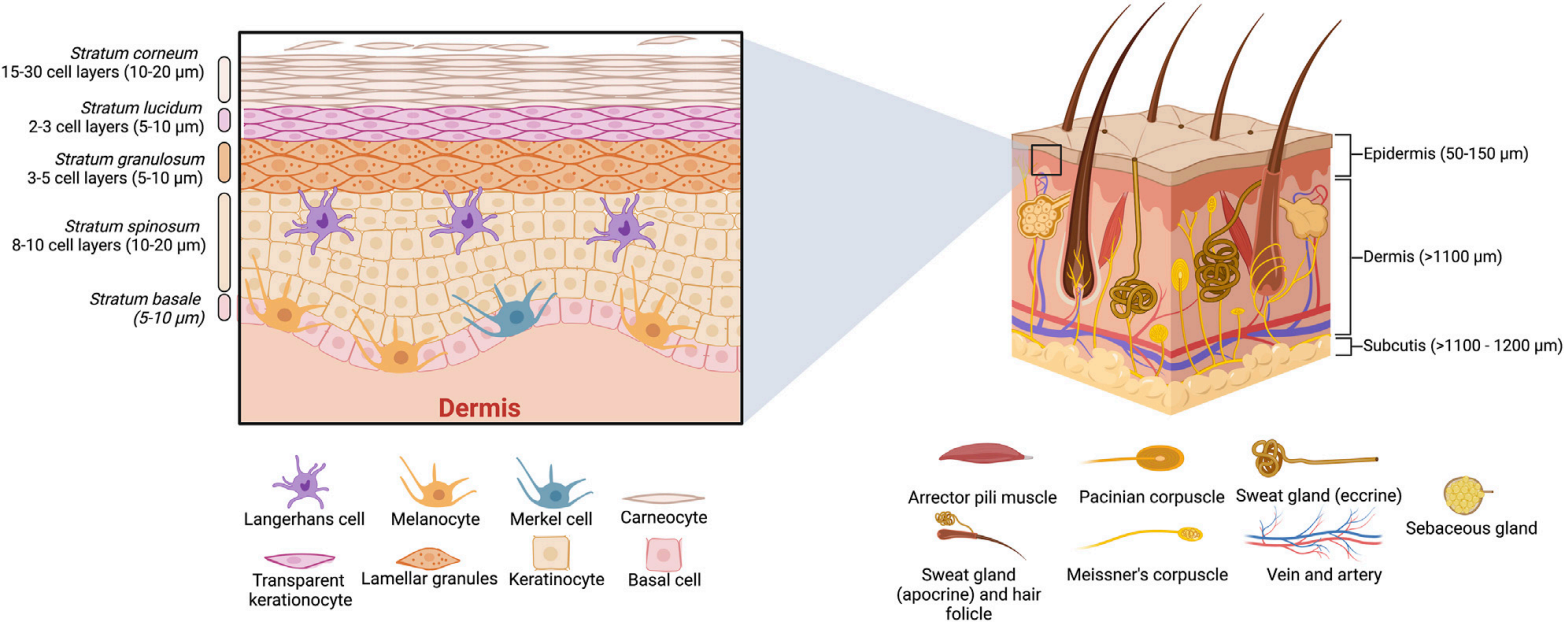
Schematic representation of the human skin anatomy, including a cross-section of layers and appendages.

Transdermal drug delivery systems (TDDS) have been explored as alternative drug delivery systems by using transdermal patches (TP). TP have been studied for many years; the first scopolamine transdermal patch was approved by the FDA in 1979 (Kang et al., 2022). Since then, TP have been studied intensively because they are less invasive than injecting drugs and have advantages over oral medications since drugs do not pass through the gastrointestinal system (Yewale et al., 2021). Furthermore, the drug delivery system by TP does not depend on the liver function for spreading through the circulatory system, allowing an improved pharmacokinetic profile and minimizing undesirable side effects (Ariamoghaddam et al., 2018; Yewale et al., 2021). These advantages promoted TDDS to become an innovative field with great potential. Nowadays, it is possible to find several TP on the market for hormone therapy, postmenstrual syndrome, contraceptives, pain, smoking cessation, etc. (Yewale et al., 2021). Only in 2022, TDDS global market was evaluated at USD 31.9 billion and is projected to rise more than USD 52 billion in 2032 with Novartis International, Pfizer Inc, Endo Pharmaceuticals, and GlaxoSmithKline as key companies in this market (Market Research Future, 2022). Nevertheless, despite the tremendous success of TP, they present limitations for drug delivery due to skin layers, mainly *stratum corneum* (Figure 1), a layer of 10–20 µm of death tissue which restricts the permeability of most of the hydrophobic and hydrophilic drugs (Henry et al., 1998; Raju et al., 2018; Phatale et al., 2022). To overcome this drawback, different strategies have been developed to enhance the permeation of drugs across the skin: iontophoresis, sonophoresis, electroporation, microneedles, microemulsions, solid lipid nanoparticles, invasomes, transferosomes, dendrimers, liposomes, and ethosomes (Carter et al., 2019; Phatale et al., 2022). Among these alternatives, microneedle patches (MP) have gained special attention recently.

MP are an advanced strategy for the safe, painless, easy, and efficient delivery of drugs across the *stratum corneum* to reach the dermis layer (Tsioris et al., 2012; Hu et al., 2021). The micro-sized needles of MP create microchannels that penetrate the different skin barriers and allow delivering of drugs into deeper layers of the skin (Alexander et al., 2012; AL-Japairai et al., 2020). However, microneedle arrays in transdermal patches are not a recent topic. In 1976, Gerstel & Place patented the first MP; however, at that time, biomaterial technology was less advanced than today (Gerstel and Place, 1976; Tsioris et al., 2012). With the development of novel biocompatible materials, fabrication methods, and characterization techniques, MP became an attractive TDDS (Yang et al., 2019; Makvandi et al., 2021; Phatale et al., 2022). At present, there are some commercially available microneedle-based products (for acne and wrinkle treatment, hair loss, delivering influenza virus vaccine, and peptides, among others) and a few MP platforms for TDDS, which are available to be loaded with drugs and have been tested in clinical trials. However, there are still no commercial MP products for treating a specific disease (Halder et al., 2021; Sartawi et al., 2022). For 2023, their market value was projected to be USD 768.9 million, which is expected to increase by two-fold in 10 years (Future Market Insights, 2023.). Such growth of the MP area is due to its versatility and ability to be used for different treatments. So far, there are many reports of MP being tested for diabetes (Rabiei et al., 2021), wound healing (Chi et al., 2020), cancer (Ali et al., 2017), dermatological diseases (Yeo et al., 2017), ocular drug delivery systems (Than et al., 2018), anti-fungal applications (Peng et al., 2021), analgesia (Chen et al., 2017), and anti-obesogenic (Liang et al., 2023).

There are several revisions about MP approaches focusing mainly on microneedle materials, microfabrication methods, delivery strategies, and biomedical applications (Yang et al., 2019; Makvandi et al., 2021; Phatale et al., 2022; Sartawi et al., 2022). On the other hand, there are few review articles focusing on MP with obesity approaches. Moreover, those reviews lack a deep analysis of all recent reports where MP with anti-obesogenic activity were tested (Wang et al., 2022; Zhu et al., 2022; Gopan et al., 2023). Therefore, due to the growing interest in the search for treatments against obesity and the potential application of microneedle transdermal patches as drug delivery system, the present work seeks to detail and summarize the state of the art of the MP with anti-obesogenic activity. To achieve this, an overview of the skin layers is described to understand their compositions and the importance of reaching certain parts of the skin to ensure the delivery of an active compound. Subsequently, the most used materials in the fabrication of MP are addressed. Then, the essential section of this work analyzes and discusses all MP reported until now (between 2017 and 2023) where anti-obesogenic compounds were immobilized, highlighting the biomaterial type, drug loading, delivery strategies, and the design of microneedle arrays. The suitability of the different materials for the anti-obesogenic drug loading onto MP, the kinetic data, and the biological effects for each case are discussed. Additionally, since pharmacology, target sites, and dosses of these tested molecules have already been intensely studied, the strategies of how these molecules were embedded into MP (liposomes, nanoparticles, free molecules, chemical modification) are described rather than the action mechanisms. Finally, perspectives and conclusions are addressed.

## 2 Targeted transdermal drug delivery

TDDS, including MP, provide a controlled delivery system with prolonged administration and a sustained effect of drugs. In contrast to conventional administration methods, such as injections and oral medications, MP offers a non-invasive, effortless, and safe usage for patients of all ages (Jeong et al., 2021). These systems are intended to overcome the specific diffusion restrictions of the different layers of the skin. Specifically, MP must be formulated to penetrate all the skin layers successfully and deliver the drug into the dermis, where the bloodstream can be found and adequately distributed (Vitorino et al., 2015). To understand the primary function of MP, it is essential to describe the different skin layers (see Figure 1).

As mentioned previously, the epidermis acts as a barrier to external substances. The epidermis has a thickness of approximately 50–150 μm, as seen in Figure 1, and it is composed of multiple layers with different degrees of cell keratinization. The outermost of these layers is called the *stratum corneum*, known as the nonviable epidermis, and represents the main challenge for drug penetration due to its barrier-like behavior. This diffusion-restricted behavior is attributed to the compact packaging between hydrophilic keratin proteins and hydrophobic lamellar lipids (Vitorino et al., 2015; AL-Japairai et al., 2020; Phatale et al., 2022). *Stratum corneum*, with a thickness of around 10–20 μm, is mainly composed of corneocytes, which are dead keratinocytes that are layered in a brick-like matrix. Under the *stratum corneum*, the next layer, the *stratum lucidum,* is a thin (5–10 μm) and translucent skin layer present in hand palms and feet soils, where cells are flattened and compacted. Then is the *stratum granulosum* layer with a thickness of about 5–10 μm, composed of keratohyalin granules (keratin, loricrin, cysteine-rich proteins, and profillagrin), which are responsible for the alignment of keratin filaments. Beneath is the *stratum spinosum* layer, constituted of keratocytes, accountable for keratin production, and Langerhans cells, with immunologic functions. Finally, the *stratum basale* layer is made of basal cells and melanocytes, responsible for the pigmentation of the skin, as well as Merkel cells, with mechanoreceptor functions (Maksimovic et al., 2014). These viable epidermis layers are relevant for many drug delivery actions like binding, metabolizing, and the active transport of substances to the dermis. The dermis is where lymphatic vessels and vascularized networks (connective tissue, sweat, and sebum glands) can be found, which allow the biodistribution of permeated drugs into the bloodstream (Vitorino et al., 2015; Carter et al., 2019; AL-Japairai et al., 2020).

The effectiveness of MP in breaking through all skin layers (epidermis), reaching the dermis, and its straightforward application have placed MP as one of the most studied TDDS. In contrast, other TDDS strategies such as iontophoresis (Green, 1996; Sachdeva et al., 2010), sonophoresis (Herwadkar et al., 2012), electroporation (Denet et al., 2004), photomechanical waves (Doukas and Kollias, 2004), electron beam irradiation, and magnetophoresis (Alexander et al., 2012) involve the use of additional equipment reducing its practicality (Yang et al., 2019). The great versatility of MP is reflected by the multiple therapeutic applications that are in clinical trials where MP are employed: acne, actinic keratosis, alopecia, cancer, diabetes, hyperhidrosis, melasma, migraine, inflammatory skin diseases, osteoporosis, pain management, scars, skin aging, vaccine delivery, vitiligo, and wrinkles (Sartawi et al., 2022). In all those applications, several kinds of microneedle materials and geometries were used depending on the needle characteristics and their required action mode (Waghule et al., 2019; Yang et al., 2019). The following section describes the different type of microneedles and their main features.

## 3 Microneedle patches (MP)

Microneedles are micro-sized needles that create microchannels, penetrating the superficial skin barrier to deliver drugs into deeper layers of the skin percutaneously (Alexander et al., 2012; AL-Japairai et al., 2020). Microneedles offer higher efficiency and user compliance, faster delivery, and can be self-administered. The first design of microneedle patches is attributed to Gerstel et al., who proposed a drug reservoir adapted for the percutaneous administration of a drug (Gerstel and Place, 1976). However, it was not until 1998 that the first microneedle patch aimed for clinical use was created. These systems’ simple development and painless usage established a precedent for applying microneedle arrays in TP to deliver a vast diversity of compounds (Henry et al., 1998).

**FIGURE 2:**
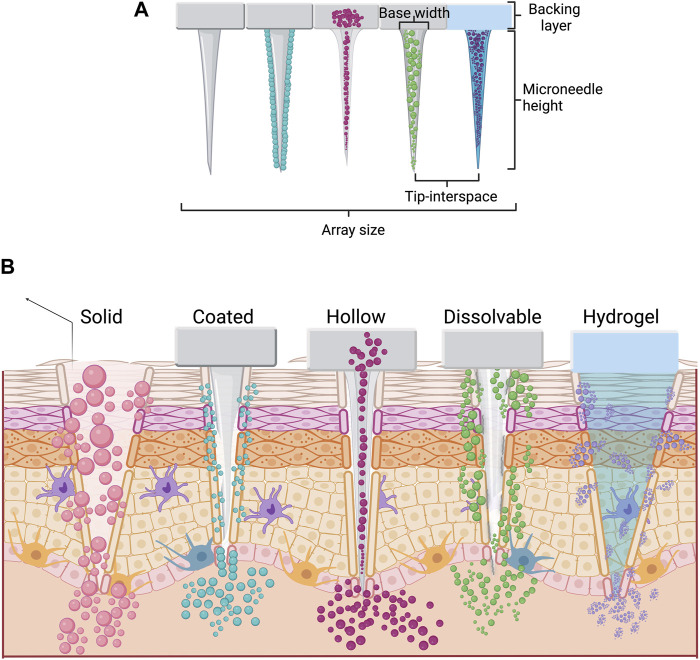
Types of microneedles in transdermal patch drug delivery. **(A)** Microneedles with essential characteristics. **(B)** Types of microneedles employed in drug delivery and their mechanism in terms of penetration in human skin.

There are several aspects that microneedle patches (MP) must assemble: biocompatibility, strong enough to pierce skin layers, proficiency in loading and releasing drugs, and, in some cases, biodegradability (AL-Japairai et al., 2020; Halder et al., 2022). The human skin tends to prevent the insertion of microneedles or lead to their breaking due to its complexity and flexibility (Kang et al., 2021; Quan, 2023). Therefore, it is mandatory to design microneedles and their array correctly. The design of microneedles considers the geometry, tip height, base width, and the distance between them (see Figure 2A) (Davidson et al., 2008; Al-Qallaf and Das, 2009). Considering these factors ensures the smooth insertion of microneedles into the skin without pain (Gill et al., 2008). Geometries used in microneedle design include rectangular with a sharp edge, conical, pointed, pyramid, bullet-shaped, cylindrical, octagonal cone, and obelisk, among others (Ghiyasi et al., 2023). The geometry of microneedles affects the drug permeability of the skin (Davidson et al., 2008). For its part, the microneedle height determines the penetration success. A distance smaller than 1,000 µm is enough to reach the dermis without pain and to deliver the drug (Han et al., 2023). Table 1 shows that the height values used in MP with anti-obesogenic application range between 500 and 1,000 µm. Spacing (tip-interspacing) and the number of microneedles affect the force needed to pierce the skin (Ghiyasi et al., 2023). Additionally, increasing the center-to-center spacing for coated microneedles improves transdermal drug delivery efficiency (Davidson et al., 2008). Currently, there are five types of microneedles, and depending on their material and design, they can be categorized as solid, coated, hollow, dissolvable, and hydrogel (Figure 2B).

**TABLE 1:** List of transdermal microneedle patches with anti-obesogenic activity. All reports used dissolving microneedles.

Microneedle material	MP characteristics	Bioactive	Molecule embedding strategy	Effect	References
HA and PLGA	- Array size: 10 × 10 - Tip-interspacing: 700 µm - Height: 600 µm - Base width: 300 µm - Shape: sharp-pointed quadrangular pyramid	β3-adrenoceptor agonist (CL 316243) and thyroid hormone T3	In solution	WAT browning and suppress gaining of body weight and iWAT and EpiWAT	Than et al. (2017)
mHA	- Array size: 11 × 11 - Tip-interspacing: 600 µm - Height: 800 µm - Base width: 300 µm - Shape: sharp-pointed conical	Rosiglitazone	Nanoparticles (dextran/alginate/rosiglitazone in solution	WAT browning, improving of insulin sensitivity, reduction of EpiWAT	Zhang et al. (2017)
HA	- Array size: 7 × 7 - Tip-interspacing: NR - Height 500 µm - Base width: NR - Tip diameter: 30 µm - Shape: cylindrical structures	Caffeine	In solution	Suppress gaining of body weight, restoring of leptin and adiponectin levels, and reduce cholesterol levels	Dangol et al. (2017)
Gelatin	- Array size: 10 × 10 - Tip-interspacing: NR - Height: 750 µm - Base width: 250 µm - Shape: Bullet	NA	NA	Reduction of SAT	An et al. (2019)
mHA	- Array size: 11 × 11 - Tip-interspacing: 600 µm - Height: 800 µm - Base width: 300 µm - Shape: sharp-pointed quadrangular pyramid	Rosiglitazone	In solution	Body weight lost and reduction of iWAT	Peng et al. (2020)
HA-PVA mixture	- Array size: 10 × 10 - Tip-interspacing: 700 µm - Height: 600 µm - Base width: 300 µm - Shape: sharp-pointed quadrangular pyramid	Capsaicin	α-lactalbumin/Capsaicin Nanomicelles in solution	Weight loss, reduction of AT, improving blood lipid levels, and adipose tissue browning	Bao et al. (2021)
PVP	- Array size: 25 × 9 - Tip-interspacing: NR - Height: 500 and 1,000 µm - Base width: 159 and 166 µm - Shape: sharp-pointed hexagonal pyramid	Liraglutide	Nanoparticles (PLGA-Liraglutide) in solution	MNP was presented as proof of concept, *in vivo* experiments were not performed	Rabiei et al. (2021)
PVA-PVP mixture	- Array size: NR - Tip-interspacing: 750 µm - Height: 800 µm - Base width: 400 µm, - Shape: sharp-pointed quadrangular pyramid	Metformin	Free and encapsulated Metformin (mesoporous BGNs) in solution	Reduction of glucose blood levels (hypoglycemic effect)	Feng et al. (2022)
PLGA	- Array size: 10 × 10 - Tip-interspacing: 500 µm - Height: 800 µm - Base width: 200 µm - Shape: sharp-pointed quadrangular pyramid	Metformin	In solution, delivery assisted by iontophoresis	WAT browning, decreasing body weight and visceral fat, increasing of energy expenditure, better glucose homeostasis, and reducing inflammation	Abbasi et al. (2022)
HA	- Array size: 10 × 10 - Tip-interspacing: 1,000 µm - Height: 650 µm - Base width: 300 µm - Shape: sharp-pointed quadrangular pyramid	Succinate	In solution	Decreasing body weight gain (Increasing lipolysis, decreasing lipogeneses) and WAT browning	Liao et al. (2022)
HA-PVA mixture	- Array size: 36 needles circular array - Tip-interspacing: 250 µm - Height: 900 µm - Base width: 300 µm - Shape: conical sharp-pointed	Capsaicin	Nanoparticles (Capsaicin -PEG-clove oil-caseinate) in solution	MP was presented as a proof of concept; *in vivo* anti-obesogenic experiments were not performed but bioavailability improvement was demonstrated	Mudhol and Serva Peddha (2023)
HA-PVA mixture	- Array size: 10 × 10 - Tip-interspacing: 700 µm - Height: 600 µm - Base width: 300 µm - Shape: sharp-pointed quadrangular pyramid	Tetradecanoic acid-2,4-dinitrophenol ester (TADNP)	Self-assembled TADNP nanomicelles in solution	Increase weight loss, reduction of blood glucose levels and WAT browning	Liang et al. (2023)

Abbreviations: MP, microneedle patches; NR, not reported; NA, not applicable; SAT, subcutaneous adipose tissue; HA, hyaluronic acid; mHA, methacrylate hyaluronic acid; PLGA, Poly (lactic-co-glycolic acid); PVA: polyvinyl alcohol; PVP: polyvinylpyrrolidone; WAT: white adipose tissue; BAT, brown adipose tissue; iWAT, inguinal white adipocyte tissue; EpiWAT, epididymal white adipocyte tissue (visceral fat); AT, adipose tissue; BGNs, bioactive glass nanoparticles.

Solid microneedles are one of the most studied types in recent years (Sartawi et al., 2022). This type of microneedles is developed using either metals (titanium, stainless steel, nickel/iron) or polymers (silicon, thermoplastic polyurethane, polydimethylsiloxane, etc.) (Jang et al., 2019; Tariq et al., 2022). When the drug is administered using solid microneedles, two steps are needed: 1) to create micro-sized holes and 2) to deliver the drug (Tariq et al., 2022). However, coated microneedles aim to eliminate the need for a two-step application. Coated microneedles have drug molecules attached on the surface, and after the MP application, the coating formulation dissolves and releases the drug into the dermis (Chen et al., 2023). The coating process can be carried out on microneedles of different materials, including metals (tungsten-alloy, stainless steel, silver, gold, copper) and silicon (Aldawood et al., 2021; Tariq et al., 2022). Furthermore, the stability of drugs may be enhanced when they are coated on the microneedle (Liang et al., 2022). Typically, coated microneedles contain low amounts of drugs (0.1–1.0 µg per needle (Kang et al., 2021); however, with an optimized design and coating solution, it could be possible to increase the drug loading capacity (Chen et al., 2023).

For its part, hollow microneedles possess a cavity inside, a bore on the tip, and a reservoir that allows the administration of microvolumes of a drug solution straight into the dermis (Cárcamo-Martínez et al., 2021). They allow the administration of accurate drug doses with controlled release time (Abd-El-Azim et al., 2023). Hollow, coated, and solid microneedles can be made of similar materials like metals (stainless steel, titanium, palladium, palladium-cobalt, nitinol, alloys, nickel), ceramics (alumina), and polymers (polyvinyl alcohol, polymethylmethacrylate, polycarbonate, etc.) (Cárcamo-Martínez et al., 2021). On the other hand, dissolving microneedles are traditionally fabricated with biodegradable and biocompatible polymers (Rabiei et al., 2021; Sartawi et al., 2022). During manufacturing, the free or encapsulated drug is mixed with the polymeric solution. After penetrating the *stratum corneum*, the polymer forming the microneedle starts its biodegradation and releases the entrapped drug (Sartawi et al., 2022). The most used polymers for dissolving microneedles are hyaluronic acid, poly (lactic-co-glycolic acid), polyvinyl alcohol, polyvinyl pyrrolidone, and gelatin (Than et al., 2017; An et al., 2019; Bao et al., 2021; Rabiei et al., 2021).

Lastly, hydrogel microneedles are the newest form; their exploration started in the early 2010s (Sartawi et al., 2022). They are fabricated with swellable hydrophilic crosslinked polymers (poly (methyl vinyl ether-alt-maleic acid), poly (methyl vinyl ether-co-maleic anhydride), poly (vinyl alcohol), poly (2-hydroxyethyl methacrylate), and silk (Turner et al., 2021). Once the microneedles are inserted into the skin, the hydrogel swells due to the presence of interstitial fluid, and then, the drug is released from the microneedle into the dermis (Turner et al., 2023). An advantage of hydrogel microneedles is that their biodegradability and drug-release kinetics can be tunable by modifying their material properties (Turner et al., 2023). This feature makes them suitable for biomedical applications, allowing their use as a minimally invasive method for diagnosis and transdermal administration of drugs (Turner et al., 2021; 2023).

## 4 Applications in obesity

MP have been used for a wide range of medical applications. However, their use for obesity treatment is still poorly explored. As can be seen in Table 1, the efforts to develop anti-obesogenic MP began 6 years ago. Up to now, the strategies for embedding anti-obesogenic molecules into microneedles are mainly two: 1) in solution, molecules are dissolved directly into microneedle polymeric solution; and 2) nanoparticles, molecules are nano encapsulated and then the nanoparticles are dissolved into polymeric solution. On the other hand, materials used for MP for anti-obesogenic applications are limited to hyaluronic acid, poly (lactic-co-glycolic acid), polyvinyl alcohol (PVA), polyvinyl pyrrolidone (PVP), and some mixtures among them. All those materials produce dissolving microneedles. This section describes in detail the different strategies used to develop MP for anti-obesogenic purposes.

### 4.1 Hyaluronic acid (HA) and HA-polymer mixtures

HA is a natural polymer composed of N-acetyl-D-glucosamine and D-glucuronic acid residues, which are repeated regularly (Snovetk et al., 2020). Table 1 shows that HA is the most used polymer in MP with anti-obesogenic activity. This agrees with the analysis of Sartawi et al. which found that HA is used in over 50% of all dissolving MP (Sartawi et al., 2022). The wide use of HA for microneedles is due to its rheological properties, which allow it to penetrate the SC and biodegrade for the controlled release of drugs into the dermis. In some cases, HA is used as a unique polymer in microneedles. However, some variants have been explored, such as methacrylated HA (mHA) or a mixture of HA with polyvinyl alcohol to enhance the mechanical properties of microneedles.

**FIGURE 3:**
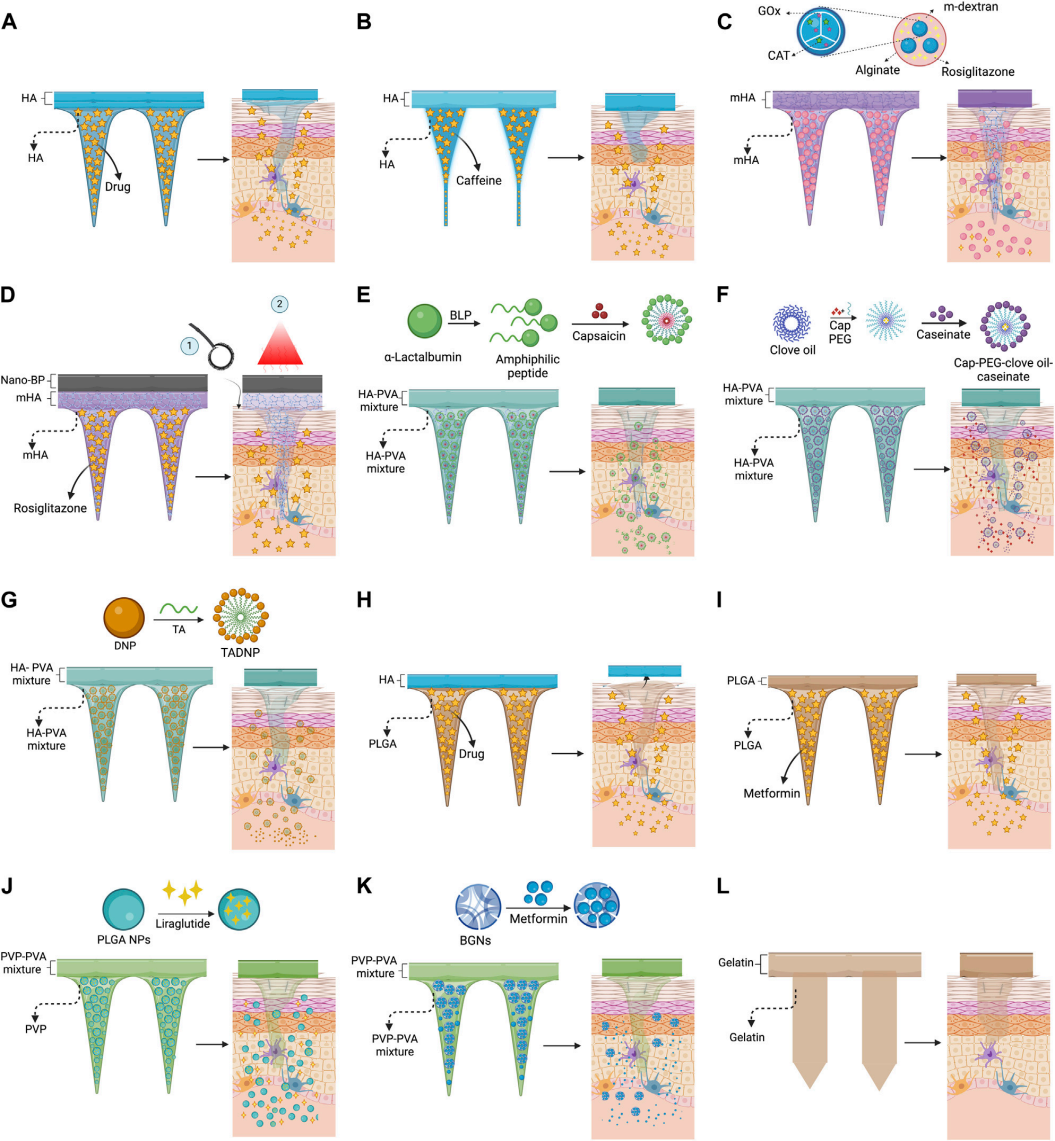
Strategies for the transdermal delivery of anti-obesogenic molecules using microneedle patches. **(A)** HA microneedles for the delivery of β3-adrenoceptor agonist (Than et al., 2017) and succinate (Liao et al., 2022); **(B)** HA microneedles (cylindric) for the delivery of caffeine (Dangol et al., 2017); **(C)** m-HA microneedles for the delivery of rosiglitazone encapsulated along glucose oxidase and catalase into m-dextran nanoparticles (Zhang et al., 2017); **(D)** m-HA microneedles with a black-phosphorus layer for the delivery of rosiglitazone assisted by metal roller and near-infrared irradiation (Peng et al., 2020); **(E)** HA-PVA microneedles for the delivery of encapsulated capsaicin into α-lactalbumin nanomicelles (Bao et al., 2021); **(F)** HA-PVA microneedles for the delivery of encapsulated capsaicin into PEG-clove oil-caseinate (Mudhol and Serva Peddha, 2023); **(G)** HA-PVA microneedles for the delivery of nanomicelles made of tetradecanoic acid-DNP ester (Liang et al., 2023); **(H)** PLGA microneedles with HA backing layer, which is detached ≈1 min after its application, for the delivery of β3-adrenoceptor agonist (Than et al., 2017); **(I)** PLGA material for microneedles for the delivery of metformin (Abbasi et al., 2022); **(J)** PVP microneedles for the delivery of liraglutide encapsulated into PLGA nanoparticles, the backing layer was made of PVP-PVA mixture (Rabiei et al., 2021); **(K)** PVP-PVA microneedles for the delivery of metformin encapsulated into bioactive glass nanoparticles (Feng et al., 2022); **(L)** gelatin microneedles without encapsulated molecules (An et al., 2019).

Than et al. developed MP by a two-step micromolding method for administering β3-adrenoceptor agonist and thyroid T3. Molecules were dissolved in HA solution (0.5 g/mL, <10 kDa), meanwhile the backing layer had the same HA composition, but without the anti-obesogenic drug (Figure 3A). The microneedles were dissolved within 2 min in mice. Than et al. demonstrated that thyroid T3 administration by MP takes more time to reach the maximum serum concentration, and, as a consequence, the half-life increases compared to intraperitoneal injection. In the same line, intraperitoneal injection of β3-adrenoceptor administration (0.5 mg kg^−1^ d^−1^) exerted a browning effect on mice´s white adipocyte tissue (WAT). However, when MP were used for transdermal administration, the same effect was observed using a concentration five times lower (Than et al., 2017). Similarly, Liao et al. used only HA to fabricate MP. In their work, a succinate solution (3.3% wt) was added to an HA solution of 30% wt (8–15 kDa) to create MP loaded with succinate (Figure 3A). In this case, microneedles and the backing layer were made of the same material. It was observed that 8 min after MP application into murine skin, microneedles showed a high degree of degradation. Also, it was observed that the application of MP loaded with succinate every 3 days for 7 weeks reduced the body weight gain by almost 3-folds after 50 days. Besides, the treatment helped reduce the adipocyte size of inguinal white adipocyte tissue (iWAT) and epididymal white adipocyte tissue (EpiWA). Finally, clear evidence suggests that MP loaded with succinate promotes the browning of WAT (Liao et al., 2022). For its part, Dangol et al. developed an HA-based MP for the transdermal delivery of caffeine but using a higher molecular weight of HA (100–200 kDa) (Figure 3B). The dissolved microneedles were produced by a lithography technique, mixing a solution of 15% caffeine and HA. It was observed that HA inhibits the crystal growth of caffeine, an inactive form of caffeine. After insertion of MP into pig skin, a considerable dissolution was observed after 30 min. In addition, more than 94% of caffeine was released within 6 hours. However, almost 100% of caffeine was detected in the caffeine receptor even after 1 day of MP application. The maximum serum level of caffeine in treated mice (38 μg/mL) was reached within 5 hours after MP application, which is higher in comparison to the control group (topical administration of caffeine). Caffeine-loaded MP reduce body weight gain and decreases serum leptin but increases adiponectin levels and reduces total cholesterol (Dangol et al., 2017).

As mentioned previously, an alternative to increase the strength and reduce the biodegradation rate of HA is the modification with methacrylate molecules to produce mHA (Poldervaart et al., 2017). Following this strategy, Zhang et al. developed a mHA-based MP for the transdermal delivery of rosiglitazone, an agonist of peroxisome proliferator-activated receptor gamma (PPARγ). They encapsulated rosiglitazone along with glucose oxidase and catalase in acidic-sensitive dextran nanoparticles covered by an alginate layer, and finally, the nanoparticles were embedded in mHA solution for a further crosslinking reaction (Figure 3C). This interesting delivery system allows glucose oxidase to decrease the local pH by converting glucose into gluconic acid. Such pH reduction allows for the controlled degradation of dextran nanoparticles and the release of rosiglitazone. At the same time, catalase consumes hydrogen peroxide produced by glucose oxidase reaction. In parallel, mHA is dissolved inside the dermis, allowing rosiglitazone to reach the adipose tissue. The mHA microneedles were completely dissolved after 3 days. However, neither complementary analysis of the mechanical strength of microneedles nor degradation kinetics was performed. Respecting the anti-obesogenic effect, WAT browning was observed after the treatment (Zhang et al., 2017). It is worth mentioning that derived from this strategy, two patents were presented and approved: WO 2019/055594 A1 and US 2020/0246321 A1. Also, using a similar approach, Peng et al. developed MP for the transdermal delivery of rosiglitazone. Unlike the previous example, Peng et al. directly mixed a rosiglitazone solution (25 μg/mL) with a mHA solution at 0.05 g/mL for further crosslinking reaction. Once the MP was produced, a layer of black phosphorus nanoparticles was added on top of the MP to promote the rosiglitazone release and, at the same time, assisted the lysis of the fat cells by near-infrared irradiation (see Figure 3D). Before the application, a metal roller microneedle was used to create micron-sized holes on the skin of C57 mice to increase the size of the drug delivery channel. For its part, infrared irradiation increases the heating rate of the MP surface to reach 40°C–45°C, increasing the delivery rate of rosiglitazone. The MP was applied every 3 days for 4 weeks. As a result, it was observed that rosiglitazone-loaded MP reduced the body mass gain and inguinal white adipocyte tissue (Peng et al., 2020).

Another strategy to improve the mechanical properties and the drug release of MP is blending HA with PVA. PVA is a biocompatible and non-ionic synthetic polymer that is biodegradable and inert under physiological conditions (Lewandowska, 2020). Using this blending approach, Bao et al. developed MP for the transdermal delivery of capsaicin. They encapsulated capsaicin into α-lactalbumin nanomicelles for further embedding into microneedles made of HA-PVA mixture (Figure 3E). α-lactalbumin nanomicelles were self-assembled due to hydrolyzed amphiphilic peptides produced by partial hydrolysis of α-lactalbumin. During the nanomicelles formation process, the capsaicin solution (1 mg/mL) was added. The α-lactalbumin nanomicelles loaded with capsaicin were lyophilized and poured into a mixture of water, HA, and PVA at a mass ratio of 17:2:1. *In vitro* analysis revealed that 98% of capsaicin was released within 50 min at pH 6.4% and 78% at pH 7.4. However, microneedles were almost completely melted within 30 min *in vivo* conditions. Besides, it was observed that transdermal administration of micellar-encapsulated compounds can increase the subcutaneous retention of small molecules like capsaicin. Concerning anti-obesogenic activity, once α-lactalbumin nanomicelles loaded with capsaicin are released from MP, nanomicelles are endocytosed into adipocytes, and capsaicin is released intracellularly to exert biological activity. It was observed that there was a substantial body mass reduction (22.4%) in high-fat diet mice treated with MP-containing encapsulated capsaicin. In addition, the α-lactoalbumin nanomicelles delivery system allowed them to obtain higher capsaicin concentrations in blood compared to MP with free capsaicin in solution. Surprisingly, α-lactoalbumin nanomicelles, without capsaicin, showed anti-obesogenic activity by promoting weight loss. Furthermore, capsaicin administration using MP produced an enhanced anti-obesogenic effect in contrast to the local injection of capsaicin. This strategy generally decreased triglycerides and total cholesterol blood levels, induced weight loss, and the browning effect of WAT (Bao et al., 2021).

Mudhol & Serva Peddha encapsulated capsaicin into clove oil-sodium caseinate nanoparticles intending to inhibit the crystal formation of capsaicin and improve the load capacity. Capsaicin-loaded nanoparticles (100–200 nm) were embedded in a mixture of PVA 10% and HA 0.5 g/mL to produce MP (see Figure 3F). Kinetics revealed that all capsaicin was released within 3 min in either *in vitro* or *ex vivo* assays. However, *in vitro* assays showed that capsaicin concentration in plasma reached its maximum within 4 hours, and then it started to decrease, but capsaicin levels were detected even 32 h after the MP application. Furthermore, the capsaicin administration using capsaicin-loaded nanoparticles embedded into MP favors the capsaicin accumulation into the adipose tissue (435 ng/g) almost 10-fold greater than the non-encapsulated capsaicin (Mudhol and Serva Peddha, 2023).

Lastly, Liang et al. developed an interesting approach using MP as an anti-obesogenic treatment. They used 2,4-dinitrophenol (DNP), a molecule that reduces the volume of white adipocytes and promotes browning effect. However, if the molecule targets different cells, a toxic effect could be observed. To confer adipose tissue targeting, DNP was esterified with tetradecanoic acid, an FDA-approved adjuvant and crucial component of adipocytes. The resulting amphiphilic tetradecanoic acid-DNP ester was self-assembled into nanomicelles (215 nm) which were further mixed with a solution of water, HA, and PVA at a mass ratio of 17:2:1, respectively, to produce MP (see Figure 3G). Using the HA-PVA mixture, it was possible to produce microneedles with enough mechanical strength (0.4 N) to pierce the skin. After 30 min of application, the MP showed substantial degradation due to body temperature. Regarding DNP biodistribution, it was observed that MP favor the accumulation of DNP into iWAT and decreases in other organs (heart, liver, and kidney). Besides, the administration of tetradecanoic acid-DNP ester nanomicelles increases weight loss, reduces blood glucose levels, and produces a browning effect on WAT (Liang et al., 2023). From the last three reports, where HA-PVP microneedles were created, it is possible to observe that the HA concentration plays an important role in the degradation rate of microneedles and, consequently, the drug-releasing time. Unfortunately, the HA molecular weight was not specified in those reports. For its part, despite the PVA concentration used by Mudhol & Peddha being higher than that used by Bao et al. and Liang et al., the degradation rate of microneedles was not improved.

### 4.2 Poly (lactic-co-glycolic acid) PLGA

PLGA is a polymer composed of repeating units of lactic and glycolic acid, which are linked by ester bonds, an easily degradable bond in aqueous solution (Marquina et al., 2023). The ratio between lactic and glycolic acid, as well as their structure, play an important role in the physical properties (solubility, mechanical strength, and degradation kinetics) of PLGA (Abbasi et al., 2022; Marquina et al., 2023). Additionally, when the proportion of lactic acid increases, the degradation rate decreases, and it could take up to 6 months for total degradation (Marquina et al., 2023). Following this approach, Than et al. used PLGA-based MP by two-step micromolding method for the administration of β3-adrenoceptor agonist and thyroid T3. They used three different types of PLGA: long chain 756S (76–115 kDa, lactide:glycolide 75:25), short chain 502 (7–17 kDa, lactide-glycolide 50:50), and small-branched, and combinations of those for microneedles synthesis. An interesting strategy was that HA molecules were placed between PLGA-microneedles and the backing layer (see Figure 3H), allowing microneedles to detach from the backing layer once interstitial fluid reaches the HA layer (≈1 min) and dissolve it. It was found that long chains of PLGA favor sharp-pointed pyramidal shapes but slow drug release (37.3% in 2 weeks). In contrast, short and small-branched PLGA could not form a well-defined morphology, but the drug release rate was faster (70% in 2 weeks). Therefore, a mixture 1:1 of long:short PLGA chain was tested giving a well-formed pyramidal shape, with a mechanical strength enough to overcome skin penetration (10 mN) and achieve a drug-release of 31.8% in 4 days and a penetration depth of ≈300 μm. It was demonstrated that β3-adrenoceptor agonist delivery by MP in obese mice could promote WAT browning, decreasing visceral fat and diminishing total cholesterol, free fatty acids, and insulin in serum levels (Than et al., 2017).

A similar approach was performed by Abbasi et al., where they used PLGA with a lactide:glycolide ratio of 50:50 to produce microneedles with embedded metformin (see Figure 3I). For the microneedle fabrication, PLGA (2000 mg/mL) was dissolved in dimethylformamide and mixed with metformin. The backing layer was made of PLGA with a lactide:glycolide ratio of 75:25 without the drug. MP had a metformin loading of 91.3 µg. In *in vitro* experiments, almost 90% of metformin was released within 8 hours, and the total metformin release was reached at 24 h. During *in vivo* assays, in addition to the natural biodegradation of PLGA for metformin release, iontophoresis was used to force metformin permeation through the dermal layer and reach subcutaneous WAT. A current of 0.2 mA/cm^2^ was applied for 30 min for iontophoresis application. The assistance of iontophoresis for metformin release helped slightly boost the anti-obesogenic effect of metformin-loaded MP. The anti-obesogenic effects observed were WAT browning, increased energy expenditure, decreased body weight and visceral fat, better glucose homeostasis, and reduced obesity-related inflammation. Since the assistance of iontophoresis showed a very similar effect to the MP application alone, and iontophoresis could not be practical for routine application at home, metformin-loaded MP itself could represent a suitable option as an anti-obesity treatment (Abbasi et al., 2022).

### 4.3 Polyvinyl alcohol (PVA) and polyvinyl pyrrolidone (PVP)

PVP is a polymer that has been employed for a long time in biomedical applications due to its biocompatibility, water solubility, chemical stability, and amphipathic behavior (Teodorescu and Bercea, 2015). Using PVP, Rabiei et al. prepared MP with embedded PLGA nanoparticles loaded with liraglutide (see Figure 3J). For its production, liraglutide-loaded PLGA nanoparticles (353 nm size and a Pdi of 0.413) were produced by the water/oil/water emulsion solvent evaporation method. The nanoparticles were mixed with a PVP solution (50% w/v) for microneedles synthesis; meanwhile, the backing layer was made of 10% w/v of a PVP-PVA mixture (this composition provided flexibility to the patch). The composition of PVP, 50% w/v, allowed a proper mechanical strength for skin penetration (4.32 N), and when PLGA nanoparticles were present, stronger microneedles were produced (5.21 N). The PVP microneedles were completely dissolved within 60 min. However, only 55% of liraglutide could reach the dermis due to only a few microneedles penetrating the skin properly. According to their calculations, an array of 308 microneedles is needed to administrate 0.6 mg of liraglutide for 1 week without replacing the MP. Despite no anti-obesogenic assays being performed, it is important to highlight that liraglutide is an FDA-approved drug for obesity treatment (Rabiei et al., 2021).

In other work, PVP was blended with PVA to enhance the mechanical properties of MP for the delivery of metformin. The FDA has not approved this drug for treating obesity; however, it has demonstrated efficacy in weight gain prevention (Pu et al., 2020). Feng et al. encapsulated metformin into mesoporous bioactive glass nanoparticles (150–200 nm), and both encapsulated metformin and free metformin were embedded into a PVA-PVP mixture for microneedles fabrication (see Figure 3K). The backing layer was made of the same PVA-PVP mixture but without metformin. The mechanical strength of PVA-PVP microneedles was enough to pierce the skin; moreover, the presence of glass nanoparticles in the microneedles increased 5-fold the toughness of microneedles. After the MP application, a swelling and degradation process started, and free and encapsulated metformin was released. Free metformin was intended for a rapid control of blood glucose levels. Meanwhile, encapsulated metformin maintains prolonged hypoglycemia (50% release of metformin was reached within 30 h). Despite the work being focused on diabetic rats and no anti-obesogenic assays being performed, metformin has proved to have anti-obesogenic effects, and therefore, this approach has a great potential for obesity treatment (Feng et al., 2022).

### 4.4 Other materials (gelatin, silicone)

Besides HA, PLGA, PVA, and PVP, other polymers have been used for MP with anti-obesogenic activity. For example, An et al. developed dissolvable gelatin MP, which produced an anti-obesogenic effect without the need to embed an additional molecule. In their work, a 10% w/w gelatin solution (from porcine or fish) was used to fabricate bullet-shaped microneedles (see Figure 3L). MP were applied every 3 days for 4 weeks. Both types of gelatins, but especially those from porcine, produced a reduction in fat accumulation. Similarly, the patent KR102085404B1 claims MP based on gelatin for partially treating obesity due to the weight loss properties (An et al., 2019).

Lastly, an interesting approach was developed by Liu et al. by submerging silicone-based MP into a solution of adipocyte phospholipase A2 siRNAs (10 μg/μL) before the transdermal application. Adipocyte phospholipase A2 (AdPLA2) is implicated in adipocyte lipolysis, and it has been demonstrated that its suppression triggers higher lipolysis rates and higher energy expenditure (Jaworski et al., 2009). Therefore, it is reasonable to visualize that AdPLA2 exerts an important role in obesity development. The administration of AdPLA2 siRNAs by MP promoted a reduction of AdPLA2 expression levels (50%) but was too strong for the one obtained by injection (70%). This work was not intended to treat obesity; instead, thyroid-associated ophthalmopathy-related proptosis. However, results showed a potential strategy for anti-obesogenic treatment after some modifications in the application strategy (Liu et al., 2021).

## 5 Perspectives and conclusion

Despite the few reports using MP as a transdermal delivery system of anti-obesogenic molecules, the results demonstrate its great potential for obesity treatment. However, some aspects must be addressed to fully understand and enhance the drug release effectiveness of anti-obesogenic molecules from MP applications.

Firstly, it is possible to modulate the drug-release kinetics by modifying the physicochemical properties of microneedle materials (Chen et al., 2023). Most of the tested materials for anti-obesogenic purposes have a quick degradation rate, and, ideally, the administration must be continuous through time. So far, only HA, mHA, PVA, PVP, and PLGA have been explored as microneedle materials for delivering anti-obesogenic molecules. However, mixtures between them (e.g., HA-PLGA, PVA-PLGA) as microneedles material could represent an interesting opportunity since blending different polymers can enhance physicochemical properties (Lewandowska, 2020). Furthermore, other biocompatible polymers such as carboxymethylcellulose, polycaprolactone, chitosan, or silk proteins (silk fibroin) are suitable polymers, used previously for MP (Liu et al., 2023), that can be explored as microneedles materials for the delivery of anti-obesogenic materials. Additionally, stimuli-responsive materials are also a suitable strategy. This approach was used by Zhang et al. by producing nanoparticles with enzymes that decrease the local pH and, consequently, the drug is released from the nanoparticle embedded in mHA microneedles (Zhang et al., 2017). Similarly, Peng et al. used a thermo-responsive MP which, using an external heat source, allowed the release of the anti-obesogenic drug (Peng et al., 2020). Stimuli-responsiveness is a strategy for drug delivery in a specific environment, increasing specificity and safety (Liu et al., 2023). Studying other stimuli-responsive systems for the transdermal delivery of anti-obesogenic drugs by MP represents an opportunity area.

The efficiency and specificity of anti-obesogenic drugs administered by MP depend on the drug’s nature and the specific delivery strategy used. However, some microneedles polymer materials like gelatin from porcine can exert an anti-obesogenic effect without loading an additional molecule (An et al., 2019). Besides, some drug delivery systems like α-lactoalbumin nanomicelles showed anti-obesogenic activity without being loaded with another molecule (Bao et al., 2021). In this context, an interesting approach to producing MP with enhanced anti-obesogenic activity could be possible using polymers and nanoparticles. In this way, an improved anti-obesogenic activity can result from the combination effect of the microneedles material, the delivery system, and the anti-obesogenic drug. For example, chitosan, chitooligosaccharides, and chitosan-derivates have demonstrated anti-obesogenic effects through different mechanisms: inhibition of PPAR-γ, stimulation of glucokinase, reduction of leptin, adiponectin, and resistin levels, inhibition of adipogenesis, lipid metabolism modulation, and suppression of phosphoenolpyruvate carboxylase (Shagdarova et al., 2023). However, their use in MP has not been explored yet. PLGA has also been demonstrated to have anti-obesogenic activity when used as an empty scaffold (Hendley et al., 2019).

On the other hand, the effect of anti-obesogenic drug concentration in MP, as well as microneedle geometry, remains unclear. All reports in Table 1 used a single concentration of the tested anti-obesogenic drug and a unique microneedle geometry. The administration of anti-obesogenic drugs by MP could achieve lower effective doses in contrast to intraperitoneal administration (Than et al., 2017). Nevertheless, analyzing the effect of drug loading content in MP is necessary to optimize the dosage without compromising the anti-obesogenic activity. Regarding microneedle geometry, the most used was the sharp-pointed quadrangular pyramid. Other microneedle shape variants, like bullet and conical shapes, were employed. It is known that the shape, interspacing distance, base width, and height have an impact on skin penetration (Tamez-Tamez et al., 2023). Following this topic, the geometry of MP will determine the effective force needed to pierce the skin. If MP are not appropriately applied, the efficiency of the drug delivery system will be compromised (Rabiei et al., 2021). Therefore, a standardization strategy for applying MP-containing anti-obesogenic molecules is needed and has not been addressed in the current reports.

After reviewing the results of MP with anti-obesogenic activity and despite this approach having been recently explored, it is possible to conclude that transdermal microneedle patches could represent a viable option to tackle obesity. As mentioned above, some important aspects must be addressed to obtain an optimized MP for obesity treatment. In the meantime, it will be crucial to test more anti-obesogenic molecules, including those FDA-approved, administered by MP to accomplish a broad portfolio of options for future obesity treatments. Unfortunately, the worldwide forecast for obesity prevalence is not favorable; 3 billion people will be diagnosed with this disease in 2025 (Lobstein et al., 2023). Therefore, the demand for obesity treatments will increase, and a large production of MP with anti-obesogenic molecules will be needed. Consequently, scalability options for large MP manufacturing must be addressed.
